# Participation in screening for breast and cervical cancer among women with current or previous drug use: a survey study

**DOI:** 10.1186/s12889-023-15236-3

**Published:** 2023-02-16

**Authors:** Lars Garpenhag, Disa Dahlman

**Affiliations:** 1 Center for Primary Health Care Research, Department of Clinical Sciences, Lund University/Region Skåne, Malmö, Sweden; 2 Division of Psychiatry, Department of Clinical Sciences Lund, Lund University, Lund, Sweden

**Keywords:** Health Services accessibility, Sweden, Cancer disparities, Women, Opioid substitution treatment, Drug use, Cancer screening

## Abstract

**Background:**

Women with current or previous drug use (WCPDU) have an increased risk of poor breast and cervical cancer outcomes. Screening is known to decrease the mortality of these common cancer forms, but screening participation has been sparsely investigated among women with drug dependency. The aim of this study was to assess participation in screening for breast and cervical cancer among WCPDU.

**Methods:**

We recruited WCPDU to a survey study, from six opioid substitution treatment (OST) clinics and one needle exchange program (NEP) in Malmö, Sweden, and through the Drug Users Union in Stockholm, Sweden. The survey was constructed according to results from focus group discussions about cancer screening in a sample of women in OST. Survey data were analyzed using descriptive statistics. We analyzed associations between non-compliance to screening and healthcare contact (OST, NEP or none) by logistic regression analysis; unadjusted and adjusted for age, native language, housing situation, educational attainment and main source of income.

**Results:**

A total of 298 women (median age 43 years) responded to the survey. The self-reported compliance with cancer screening recommendations was 29% for breast cancer screening and 41% for cervical cancer screening. Non-compliance with cervical cancer screening was associated with NEP participation in univariate but not multivariate analysis. We did not find an association between non-compliance with breast cancer screening and healthcare contact. Non-compliance with screening for cervical cancer was also associated with unstable housing in univariate and multivariate analyses, and inversely associated with increasing age in a univariate analysis. Non-compliance with breast cancer was associated with unstable housing in a univariate analysis, and inversely associated with not having Swedish as a native language in a multivariate analysis.

**Conclusion:**

The self-reported compliance with the national cancer screening programs for breast cancer and cervical cancer of WCPDU is notably lower than in the Swedish general population. Women with unstable housing seem to be particularly vulnerable to non-compliance with cancer screening. Interventions to minimize barriers to cancer screening are crucial to decrease the increased cancer morbidity and mortality among WCPDU.

**Supplementary Information:**

The online version contains supplementary material available at 10.1186/s12889-023-15236-3.

## Background

Breast cancer and cervical cancer are two of the most common forms of cancer diagnosed in women globally [1], causing significant mortality and morbidity worldwide. Many countries, including Sweden, provide population-based screening programs for early detection and successful treatment of these cancer forms [2, 3].

The risk of poor breast and cervical cancer outcomes is not evenly distributed throughout the population. Recent Swedish total-population studies have shown that women with current or previous drug use (WCPDU) have an increased risk of metastasized and fatal breast cancer [4], as well as incident – but not fatal – cervical cancer [5]. The discrepancy between the risk increase of breast cancer incidence and mortality in WCPDU was interpreted as a consequence of poor screening compliance and delayed healthcare seeking behavior [4]. Finnish data has shown an increased breast cancer mortality among women with substance use disorders (SUD) including but not limited to illicit drugs [6], and one Australian study found an elevated risk to die from cervical cancer, but not breast cancer, in women with opioid dependence [7]. Australian studies have also shown that WCPDU have an increased risk of incident cervical cancer and cervix cytological abnormalities [8–10], which might be related to behaviors associated with increased risk of cervical cancer that are also common among WCPDU, such as smoking [11] and risky sexual behaviors [12, 13].

While the results above indicate the crucial importance of screening attendance among WCPDU, this topic has been sparsely investigated. Previous studies have shown lower rates of cervical cancer screening among WCPDU, including both women in opioid substitution treatment (OST) and women who currently use illicit drugs [9, 14, 15]. Associations have also been found between SUD and non-participation in breast cancer screening [16]. In addition, a growing body of research shows that unmet healthcare needs and lacking access to healthcare – including preventive care – are highly prevalent issues among people who use drugs [11, 17–20].

Breast and cervical cancer screening among WCPDU have, to the best of our knowledge, never been assessed in a Swedish setting, where screening is free of charge and coverage of screening programs is high overall; approximately 80% of women of relevant ages comply with the screening recommendations for cervical cancer and 70% comply with the screening recommendations for breast cancer [21]. The aim of this study was to assess participation in screening and compliance with screening guidelines for breast and cervical cancer among Swedish WCPDU, and to investigate determinants of screening participation. Our definition of WCPDU included women with current or previous illicit drug use, including non-medical use of prescription drugs.

## Methods

This survey study was part of a larger mixed-methods project on cancer screening participation among Swedish WCPDU, following an overarching sequential design where a preceding qualitative focus group study (interviews initiated in 2020, paused due to the Covid pandemic, and concluded in the fall of 2021) was conducted as part of preparations for the current one (conducted in late 2021 and early 2022) [22]. While the greater project concerns participation in breast and cervical cancer screening as well as barriers and facilitators to access of screening services, this paper focuses solely on questions concerning levels of participation. The project has been approved by the Swedish Ethical Review Authority (file no. 2020–04150).

### Setting

The study was conducted in two Swedish cities; Stockholm (ca. 1.5 million residents) and Malmö (ca. 350,000 residents). Stockholm is the Swedish capital and Malmö is the third largest city in Sweden, located in Skåne county in southern Sweden.

Sweden is a country with high drug-induced mortality, approximately four times higher than the European average [23], and a traditionally restrictive drug policy [24]. Physical and psychiatric healthcare, including OST and NEP visits, is covered by universal health insurance and thus tax-financed and strongly subsidized for the individual patient [25]. There are public as well as private providers of OST, while all the NEPs have public providers. Women who use drugs can receive medical help from psychiatric care units including OST units, and NEPs. Enrolment in OST is open to people 20 years of age or older (exceptions can be made) who have had opioid dependence for at least one year, while patients have to be 18 years old to be eligible for NEP enrolment. Outside the healthcare system, WCPDU can receive assistance from social services, peer support groups and volunteer organizations that cater for people with SUD and/or psychosocial difficulties.

Sweden’s nationally mandated population-based cancer screening programs are based on recommendations from The National Board of Health and Welfare that are implemented by the county councils on the regional level. At the time of data collection, regular screening at certain intervals (see Table 1) was recommended for female residents aged 23–64 for cervical cancer [26] and aged 40–74 for breast cancer [27]. Screening is actively offered free of charge to women in relevant age groups through invitation via mail. The mortality of cervical cancer has decreased from 8/100,000 to 2/100,000 inhabitants and the incidence has more than halved since the introduction of screening [28], while screening for breast cancer via a mammogram is estimated to have reduced the mortality rate by at least 16–20% depending on age group [27].

### Participants

Women were recruited to the study from six of seven OST clinics operating in Malmö when the study was conducted (three public and three private clinics) and the NEP in Malmö (two of the OST clinics are run by the same company, and they invited us to conduct the study at only one of them). The intent was to offer every female patient the opportunity to participate in the study. In Stockholm, we recruited WCPDU through the non-profit peer support organization Stockholm Drug Users’ Union (DUU). With the DUU’s help, we were able to reach a broader population consisting both of women enrolled in OST, women participating in NEPs, and women who had ongoing contacts with neither type of service – the latter being a group that would have been very difficult to reach in Malmö. The DUU recruited participants among female visitors to their own office, as well as at healthcare facilities (including OST clinics and NEPs) and other organizations meeting WCPDU. See Table 1 for information concerning the recruitment sites.

The inclusion criteria were for participants to be aged 23 and older (since cancer screening is not recommended to women younger than 23 years of age in Sweden), and participation in OST or NEP in Malmö, or contact with organizations collaborating with the DUU in Stockholm. Exclusion criteria were severe psychiatric conditions, language barriers or drug influence hindering the individual from giving informed consent. Women who were excluded on one occasion were allowed to participate at a later date - if the circumstances motivating exclusion were no longer present. All individuals received oral and written information about the study and provided written informed consent prior to study participation. Recruitment was conducted by OST and NEP staff in Malmö, and DUU staff and their network in Stockholm, who also were on hand to assist persons experiencing difficulties completing the form. The study participants received a grocery store gift voucher valid for 100 Swedish crowns (ca. USD $12).

**Table 1:** Clinics and organizations involved in study participant recruitment

Recruitment site	Type of clinic/organization	City
Site 1	OST clinic	Malmö
Site 2	OST clinic	Malmö
Site 3	OST clinic	Malmö
Site 4	OST clinic	Malmö
Site 5	OST clinic	Malmö
Site 6	OST clinic	Malmö
Site 7	NEP	Malmö
Site 8	NGO (DUU’s office)	Stockholm
Site 9^1^	OST clinic	Stockholm
Site 10^1^	OST clinic	Stockholm
Site 11^1^	OST clinic	Stockholm
Site 12^1^	NEP	Stockholm
Site 13^1^	NGO (outreach organization)	Stockholm
Site 14^1^	Rehabilitation facility	Stockholm
Site 15^1^	Rehabilitation facility	Stockholm

OST = opioid substitution treatment. NEP = needle exchange program. NGO = non-governmental organization. DUU = Drug users’ union. N/A = not available.

1. This clinic/organization assisted the Drug users’ union in Stockholm with the recruitment of study participants.

### Survey development

The questionnaire consisted of 14 questions regarding demographic and socioeconomic factors (healthcare contact, age, native language, educational level, housing situation and main source of income), cancer screening participation (knowledge of programs and time since last completed screening of each kind, if applicable), and barriers to and facilitators of screening compliance (barriers and facilitators not analyzed here). The survey was in Swedish. For an English translation, please see the Supplementary file.

The survey was created by authors L.G. and D.D., based upon the (so far) unpublished findings by the authors from a pilot focus group interview study with women in OST. A total of 11 women were interviewed in three focus groups about their experiences, beliefs and attitudes associated with screening for breast and cervical cancer. Data from the interviews were used in the design of the part of the questionnaire that covers barriers, facilitators and interventions. The survey instrument was developed in dialogue with representatives for WCPDU and staff working closely with the target population (OST and NEP staff, staff at the Ambulatorium – a collaboration between medical and social services providing maternal care services to WCPDU – and representatives for the DUU in Stockholm). The staff and WCPDU representatives approved a preliminary version of the survey instrument before it was finalized and distributed to potential study participants.

### Survey data management

#### Outcome

Data on self-reported cervical cancer screening participation was requested from all survey participants. Compliance with cervical cancer screening recommendations was defined as self-reported screening less than three years ago for women aged 23–49 years old, and less than seven years ago for women aged 50–64 years old. Due to Swedish recommendations, data on self-reported breast cancer screening participation were requested from women aged 40 years and older only. Compliance with breast cancer screening was defined as self-reported screening less than two years ago.

Non-compliance with cancer screening was defined as all responses that were not defined as ‘compliance’. Non-compliance included two categories: Overdue screening, defined as having participated in screening but not within the recommended time span; and No lifetime screening, defined as having never been to a screening or being uncertain whether one has ever participated in a screening.

#### Exposure

The exposure variable was type of healthcare contact. The rationale behind focusing on healthcare contact was to identify sub-groups of WCPDU where interventions aiming at increased screening participation should be prioritized. We considered OST participation as a proxy variable for severe opioid dependence (which might be associated with non-compliance with preventive health services) but also for continuous healthcare contact and a certain extent of stability. NEP participation was considered a proxy variable for active injection drug use and thereby severe social marginalization. Women with neither OST nor NEP contact might be suffering from severe SUD but might also use drugs only sporadically, e.g. for recreational purposes.

Healthcare contact was a multiple-choice question in the survey with the response options “OST”, “NEP” and “neither OST nor NEP”. We included all women who had responded “OST” as OST participants, regardless if they had also reported NEP participation. The rationale for this was that we wanted to be able to compare women with continuous treatment in specialized psychiatric care (OST) with other women (in NEP or neither OST nor NEP).

#### Covariates

In order to include more precise socioeconomic data in the analyses, we adjusted the analyses for age, native language (as a determinant of healthcare access, through literacy of the Swedish healthcare system), housing situation, educational attainment and main source of income. Data regarding housing situation, educational attainment and main source of income had multiple-choice answers in the survey that were dichotomized prior to statistical analysis. Housing situation was recoded to “unstable” if the respondent replied “unstable housing” or “homeless”. Educational attainment was recoded to “10 < years” if the respondent replied “high school (10–12 years)” or “higher education (more than 12 years)”. Main source of income was dichotomized from the multiple-choice options “employment”, “temporary or permanent sick leave”, “financial support from social services”, “old age pension” and “other” into “stable main source of income” (including employment, temporary or permanent sick leave and old age pension) and “unstable main source of income” (including financial support from social services and other).

### Analysis

Analyses were conducted using SPSS Statistics Version 25 [29]. Factors associated with the outcome variables non-compliance with screening for breast cancer and cervical cancer, respectively, were analyzed by unadjusted and adjusted logistic regression analysis. The exposure variable was type of healthcare contact (‘NEP vs. OST’ and ‘none vs. OST’). The multivariate analyses were adjusted for age, native language, housing situation, educational attainment and main source of income. In order to ensure that there was no multicollinearity between the variables in the logistic regression analysis, we conducted a correlation analysis of the exposure variable and all covariates and accepted a correlation less than 0.7. P < 0.05 was considered statistically significant.

## Results

### Sample characteristics

We received 310 survey responses. Of these, n = 12 were duplicate submissions, leaving 298 individual responses. We handled duplicate responses by consequently including the first response and excluding the latter one. The responders’ median age was 43 years, ranging from 23 to 77 years (Table 2). The sample was evenly distributed between Malmö (49%) and Stockholm (51%). More than half of the sample (55%) reported OST participation, 19% NEP participation; and 26% reported contact with neither OST nor NEP. A majority (88%) had Swedish as their native language, 55% had 10 years or more of education, and unstable housing was reported by 28%. More than half (57%) reported unstable main source of income, with financial support from social services accounting for most cases.

**Table 2:** Sample characteristics. N = 298

Characteristic	n (%)	Years (range; IQR)
Median age		43 (23–77; 36–55)
Geographical region		
Malmö	147 (49.3)	
Stockholm	151 (50.7)	
Healthcare contact		
OST	163^1^ (54.7)	
NEP	57 (19.1)	
None	78 (26.2)	
Native language		
Swedish	262 (87.9)	
Other than Swedish	35 (11.7)	
*Missing*	*1 (0.3)*	
Educational level		
0–9 years	131 (44.0)	
10 < years	164 (55.0)	
*Missing*	*3 (1.0)*	
Housing situation		
Stable	214 (71.8)	
Unstable or homeless	83 (27.9)	
*Missing*	*1 (0.3)*	
Main source of income		
Stable (employment, temporary or permanent sick leave, retirement)	127 (42.6)	
Unstable (financial support from social services, other)	170 (57.0)	
*Missing*	*1 (0.3)*	

OST = Opioid substitution treatment. NEP = Needle exchange program. IQR = Interquartile range.

1. Including n = 25 who reported both OST and NEP participation.

### Participation in screening for cervical cancer and breast cancer

A total of 285 survey responders were aged 23–64 years old, and thereby included in the screening program for cervical cancer. A total of 81% of the sample reported that they knew the reason for cervical cancer screening (Table 3). Less than half of the sample (41%) reported full compliance with the recommended screening program, and 32% reported total absenteeism from or lacking knowledge about participation in cervical cancer screening. In the total sample, 57% reported non-compliance with the recommended screening intervals (Table 3). The percentage who reported non-compliance with cervical cancer screening was 52% among women in OST, 68% among NEP participants and 63% among women with neither OST nor NEP contact (Table 4).

A total of 182 survey responders were aged 40–74 years old, and thereby included in the national screening program for breast cancer. Of these, 87% reported that they knew the reason for breast cancer screening. Less than one-third (29%) reported full compliance with the recommended screening program, and 34% reported total absenteeism from or lacking knowledge about participation in breast cancer screening. Non-compliance with breast cancer screening was reported by 70% of all women of relevant ages (Table 3), 65% among women in OST, 77% among NEP participants, and by 73% among women with neither OST nor NEP contact (Table 5).

**Table 3:** Self-reported participation in screening for cervical cancer and breast cancer

Characteristic	Cervical cancer: Age 23–64 years n (%)	Breast cancer: Age 40–74 years n (%)
N included by national screening recommendation	285 (100.0)	182 (100.0)
Knowledge of reason for cancer screening		
Yes	231 (81.1)	161 (88.5)
No	51 (17.9)	16 (8.8)
*Missing*	*3 (1.1)*	*5 (2.7)*
Compliance with recommended cancer screening^1^		
Full compliance	116 (40.7)	52 (28.6)
Non-compliance	164 (57.5)	127 (69.8)
Overdue screening	72 (25.3)	65 (35.7)
No lifetime screening, or uncertain	92 (32.3)	62 (34.1)
*Missing*	*5 (1.8)*	*3 (1.6)*

1. Cervical cancer: Every 3rd year for women 23–49 years old; every 7th year for women 50–64 years old. Breast cancer: Every 18–24th month for women 40–74 years old.

### Correlates of non-compliance with screening for cervical cancer and breast cancer

In univariate analysis, self-reported non-compliance with cervical cancer screening was associated with NEP participation (OR = 2.02, 95% CI = 1.05–3.87) but not with either OST or NEP contact (Table 4). This association did not remain in multivariable analysis adjusted for age, native language, housing, education and main income. We found an inverse association between non-participation in cervical cancer screening and higher age (OR = 0.97, 95% CI = 0.95–0.99) in univariate but not in multivariate analysis. Unstable housing was associated with non-compliance in univariate (OR = 4.96, 95% CI = 2.61–9.40) and multivariable (AOR = 4.12, 95% CI = 2.08–8.16) analyses. We did not find any associations between screening non-compliance and native language, educational attainment, or main source of income.

**Table 4:** Factors associated with non-compliance to cervical cancer screening. Univariate and multivariate logistic regression. N = 285

Covariates	Non-compliance with screening	Univariate analysis	Multivariate analysis
	n (%)	OR (95% CI)	AOR (95% CI)
Healthcare contact			
OST (indicator variable)	81 (51.6% of n = 157)	N/A	N/A
NEP (vs. OST)	38 (67.9% of n = 56)	2.02 (1.05–3.87)*	1.33 (0.65–2.73)
None (vs. OST)	45 (62.5% of n = 72)	1.56 (0.88–2.78)	1.42 (0.74–2.71)
Age in years	N/A	0.97 (0.95–0.99)*	0.98 (0.95-1.00)
Not Swedish native language	23 (67.6% of n = 34)	1.74 (0.80–3.82)	1.88 (0.81–4.35)
Unstable housing	66 (79.5% of n = 83)	4.96 (2.61–9.40)***	4.12 (2.08–8.16)***
Education less than 10 years	72 (57.1% of n = 126)	1.03 (0.64–1.66)	0.93 (0.55–1.58)
Unstable main income	104 (61.2% of n = 170)	1.59 (0.98–2.58)	1.21 (0.71–2.05)

OR = Odds ratio. AOR = adjusted Odds ratio. CI = confidence interval. N/A = not applicable. * p < 0.05. *** p < 0.001.

Non-compliance with breast cancer screening was not associated with type of addiction healthcare contact in univariate or multivariate analyses (Table 5). In univariate analysis, non-participation in breast cancer screening was associated with unstable housing (OR = 2.80, 95% CI = 1.10–7.15). This association did not remain in multivariate analysis. In multivariate analysis, however, we found an inverse association between screening non-compliance and other native language than Swedish (AOR = 0.32, 0.11–0.90). No associations between non-compliance with breast cancer screening and age, educational attainment or main source of income were found.

**Table 5:** Factors associated with non-compliance with breast cancer screening. Univariate and multivariate logistic regression. N = 182

Covariates	Non-compliance with screening	Univariate analysis	Multivariate analysis
	n (%)	OR (95% CI)	AOR (95% CI)
Healthcare contact			
OST (indicator variable)	62 (65.3% of n = 95)	N/A	N/A
NEP (vs. OST)	24 (77.4% of n = 31)	2.07 (0.77–5.56)	2.09 (0.73–6.05)
None (vs. OST)	41 (73.2% of n = 56)	1.51 (0.72–3.17)	1.31 (0.59–2.91)
Age in years	N/A	0.98 (0.94–1.03)	1.00 (0.95–1.04)
Not Swedish native language	12 (57.1% of n = 21)	0.50 (0.20–1.28)	0.32 (0.11–0.90)*
Unstable housing	34 (82.9% of n = 41)	2.80 (1.10–7.15)*	2.35 (0.87–6.35)
Education less than 10 years	55 (72.4% of n = 76)	1.20 (0.62–2.33)	1.09 (0.54–2.18)
Unstable main income	73 (76.8% of n = 95)	1.84 (0.96–3.54)	1.94 (0.94–4.01)

OR = Odds ratio. AOR = adjusted Odds ratio. CI = confidence interval. N/A = not applicable. * p < 0.05.

## Discussion

This study, which is one of the first to examine self-reported participation in screening for cervical and breast cancer among WCPDU, showed low screening compliance for both cancer forms. A notable quota of the study sample reported that they had never – or were not aware of ever having – been to a cervical cancer screening (32% of women of relevant ages) and breast cancer (34% of women of relevant ages).

The low compliance with cervical cancer screening is coherent with international studies showing lower rates - in comparison to the general population, or to controls - of cervical cancer screening among WCPDU, including both women in OST and women who currently use illicit drugs, [9, 14, 15]. While there is less evidence available regarding WCPDUs’ compliance with breast cancer screening, Connecticut (US) women in OST have been found to utilize it to a much lesser degree than US women in general [15]. Widening the view to physical and preventive healthcare in general, both Swedish and international research has shown that people who use drugs underuse such services [11, 17–20].

The compliance rate in our sample is dramatically lower than in the general Swedish population for cervical cancer screening (41% in our sample vs. approximately 80% in the general population) as well as breast cancer screening (29% in our sample vs. approximately 70% in the general population) [21]. In Sweden there are, to the best of our knowledge, no previous studies on screening compliance among WCPDU. However, our results should be viewed in the light of previous Swedish evidence that pointed to a strong socioeconomic element in cancer screening compliance, suggesting that vulnerable or marginalized women are not reached by the screening programs to the same degree as others. For instance, one recent study found that factors such as low income, low level educational attainment, dependence on social assistance and benefits and non-Nordic European background negatively affected compliance with the program for breast cancer screening [30], while national statistics based on neighborhood data have shown that participation in the cervical cancer screening follows an income gradient [21]. In contrast, we have not found any associations between screening participation and education level or income. Most likely, this is an effect of the generally low socioeconomic status of our sample thus making it difficult to identify statistically significant differences within the sample.

In our selected sample of women with generally poor socioeconomic status, certain variables were associated with non-compliance with cancer screening. In a univariate analysis, non-compliance with cervical cancer screening was associated with NEP participation and unstable housing, and inversely associated with higher age. The association with unstable housing remained in multivariate analysis, while the associations with NEP participation and age did not, which suggests that these results were explained by the difference in participants’ characteristics. Non-compliance with breast cancer screening, meanwhile, was only associated with unstable housing in univariate analysis. This association did not remain in multivariate analysis, but we found an inverse association with having a native language other than Swedish.

Unstable housing can act as a barrier to participation in screening in multiple ways. In the qualitative study we conducted as preparation for the survey, several participants spoke about homelessness and transient living conditions making it difficult to receive invitations, while the ability to maintain basic hygiene was mentioned as an important precondition for participation in screening examinations as well (unpublished data). Previous research on the use of preventive health services in homeless populations has pointed to similar problems impeding utilization [31, 32]. Other issues that have been acknowledged in this context include stigma [31], and not least the compounded pressure of practical issues that homeless individuals face that compete with and may take precedence over healthcare utilization, especially of services that could be considered discretionary [33].

The relationship between non-compliance with cervical cancer screening guidelines and NEP participation could be explained by the fact that women who attend NEP, but not OST, lack the regular contact with healthcare that OST enrolment entails. This is supported by previous findings highlighting the impact that having a regular source of care have on screening rates in marginalized populations [34, 35]. It could also be that participation in OST brings greater stability in life more generally. The fact that higher age was associated with greater compliance, meanwhile, might be an effect of younger women perceiving the risk for cancer as a less pressing matter than those who are older. This finding might also be a result of healthcare providers more actively encouraging older women to attend cancer screening. Our finding that having a native language other than Swedish increases the chance of compliance with breast cancer screening is more difficult to interpret and requires further investigation.

While the reasons for poor screening compliance need to be further investigated, it could be hypothesized that psychiatric comorbidities and periods of active drug use complicate compliance with booked appointments among WCPDU. Previous qualitative findings from Malmö, Sweden, have shown that OST patients express reluctance towards healthcare contacts other than OST and NEP due to fear of stigmatizing treatment [36, 37]. While poor health literacy has been shown to affect participation in screening for cervical cancer [38] and breast cancer [39], a surprisingly high percentage of women in our sample reported that they felt knowledgeable about the reasons for cervical (81%) as well as breast cancer (89%) screening. As no objective measures of screening knowledge were applied, it cannot be ruled out that the study participants were overestimating their knowledge. Furthermore, subjectively sufficient knowledge about the screening programs does not necessarily indicate sufficient health literacy overall. Since the invitation to cancer screening is sent by mail in Sweden, unstable housing and transient living conditions might constitute a barrier to receiving invitations. This hypothesis is supported by our findings that unstable housing was associated with non-compliance with cervical cancer screening (in uni- and multivariate analysis) and breast cancer screening (in univariate analysis).

This study has limitations. First, all data regarding screening participation was self-reported. However, previous research assessing the accuracy of self-reported healthcare utilization in similar populations suggests a reasonable to substantial level of concordance with clinical data [40, 41]. In addition, in the case of mammograms and Pap smears, it has been shown that over-reporting is a greater problem than under-reporting [42]. In order to minimize recall bias, we asked the study participants only about the last time they had been to a cancer screening. Finally, while we asked participants about their utilization of NEP and OST services, we did not gather information about any other healthcare contacts. However, it is possible that ongoing contacts, e.g., with psychiatric services might have affected their screening compliance as well.

The representativity of the results in this study is somewhat difficult to evaluate, since there are no registered data on the number of WCPDU in Malmö or Stockholm. There are no data available on the number of women attending Malmö NEP during the study period. In Stockholm, the DUU staff recruited study participants through, e.g., outreach work in streets and rehabilitation facilities. Therefore it was not possible to estimate the percentage of potential study participants who answered the survey. However, at the OST clinics in Malmö that assisted with recruitment, approximately 80% of potential study participants answered the survey. It is possible that individuals who met the exclusion criteria (inability to provide informed consent due to severe psychiatric problems, severe substance influence or poor Swedish skills) have even lower screening compliance than the study participants. In order to minimize selection bias, women who were excluded on one occasion were allowed to participate at a later date - if they were able to provide informed consent.

Several factors affect the generalizability of our results. First, they must be read in the light of healthcare in Sweden being strongly subsidized, with a comprehensive primary healthcare system. Second, both Region Skåne and Stockholm are characterized by well-functioning NEP and comprehensive OST coverage. Furthermore, both Malmö and Stockholm have university hospitals and easily accessible emergency, secondary and primary care. In addition, there is a long tradition in Malmö of harm reduction interventions. The findings might therefore not be transferable to either settings abroad or other regions in Sweden where the access to substance use and other healthcare services is lower. Our sample was notably smaller than the samples used in previous survey studies on determinants of cancer screening attendance among Swedish women [43–44]. This reflects that the study concerns a population that is vulnerable and hard to reach in comparison with the population at large, and we found the sample size sufficient for the aims of the study.

Our results, showing poor screening compliance and a high percentage of total absenteeism from cancer screening, have important clinical implications. Since non-compliance with cancer screening is a well-known risk factor for poor cancer outcomes, interventions to increase screening compliance and early cancer detection among WCPDU are thus of great clinical importance and should be taken into consideration by healthcare workers and policymakers. Our findings indicate that WCPDU with unstable housing and WCPDU who participate in a NEP are particularly vulnerable to poor screening compliance, suggesting that these sub-groups should be prioritized for easily accessible cancer screening.

## Conclusion

The self-reported compliance with the national cancer screening programs for breast cancer and cervical cancer of WCPDU is notably lower than in the Swedish general population. Women with unstable housing seem to be particularly vulnerable to non-compliance with cancer screening. Interventions to minimize barriers to cancer screening are crucial to decrease the increased cancer morbidity and mortality among WCPDU.

## Electronic supplementary material

Below is the link to the electronic supplementary material.

Supplementary Material 1

## Data Availability

The SPSS data used to support the findings of this study are restricted by the Regional Ethics Board, Lund, Sweden, in order to protect people’s privacy. Data are available from Disa Dahlman, disa.dahlman@med.lu.se, for researchers who meet the criteria for access to confidential data.

## List of abbreviations

AOR: Adjusted Odds ratio
CI: Confidence interval
DUU: Drug Users Union
NEP: Needle exchange program
OR: Odds ratio
OST: Opioid substitution treatment
SUD: Substance use disorders
WCPDU: Women with current or previous drug use
