# Supplementary material for: Participation in screening for breast and cervical cancer among women with current or previous drug use: a survey study
Source: BMC Public Health. 2023 Feb 16;23:352. doi: 10.1186/s12889-023-15236-3 (PMC9936631; doi:10.1186/s12889-023-15236-3)
Supplement: Supplementary file 1 — Supplementary Material 1 [file 12889_2023_15236_MOESM1_ESM.doc]

**Questionnaire**

**1. How old are you?** __________ years old

NB! You have to be at least 23 years old to participate in the study.

**2. Do you attend OST or participate in a needle exchange program?**

[ ] Yes, OST

[ ] Yes, a needle exchange program

[ ] No, neither

**3. Is Swedish your native language?**

[ ] Yes

[ ] No

**4. What is your current housing situation?**

[ ] Housed (own permanent residence)

[ ] Unstable accommodation (temporary solutions)

[ ] Homeless (in a shelter/sleeping rough)

**5. For how long have you attended school?**

[ ] Elementary school (9 years or less)

[ ] Highschool (10-12 years)

[ ] Higher education (more than 12 years)

**6. What is your main source of income?**

[ ] Employment

[ ] Sickness benefits or pension

[ ] Welfare benefits

[ ] Old age pension

[ ] Other

**Below are four questions about screening for cervical cancer, a so called Pap smear.**

**7. Are you confident that you know why women aged 23–64 are invited to regular screening for cervical cancer?**

[ ] Yes

[ ] No

**8. Have you attended screening for cervical cancer?**

[ ] Yes, no longer than 3 years ago

[ ] Yes, 3–7 years ago

[ ] Yes, more than 7 years ago

[ ] No

[ ] I don’t know

NB! The questions below (9 and 10) are **not** to be completed if you replied “Yes, no more than 3 years ago” to question 8.

**9. For what reasons have you missed out on screening for cervical cancer? You can indicate several alternatives.**

[ ] I was afraid to find out that I have cancer

[ ] I don’t believe that cancer happens to me

[ ] I don’t care if I have cancer

[ ] I don’t feel worthy of using healthcare resources

[ ] I don’t have time, due to my addiction

[ ] I prioritized other things

Please specify:______________________________

[ ] I have bad experiences of healthcare services

[ ] I feel persecuted/stigmatized when I visit healthcare services

[ ] I feel that the screening procedure in itself is uncomfortable

[ ] I haven’t received an invitation

[ ] I didn’t comprehend the invitation

[ ] I forgot, or missed the appointment by mistake

[ ] I didn’t have the opportunity to prepare (e.g. maintain hygiene)

[ ] I couldn’t afford transport

[ ] I didn’t want to attend while under the influence

[ ] Other:_______________________­­­­­­­­­­___________________

**10. Is there anything that could make it easier for you to attend cervical cancer screening? You can indicate several alternatives.**

[ ] Social or psychological support

[ ] Practical support, to remember the time and place of appointments

[ ] The opportunity to attend screening at a needle exchange clinic, OST clinic or other addiction care service

[ ] The opportunity to attend screening at a special clinic for women with drug dependency

[ ] Digital invitations, instead of in the mail

[ ] Better invitation design
Please explain how:______________________________

[ ] Other:______________________­­­____________________

If you are younger than 40 years old, the questionnaire is finished.

If you are 40 years old or older, please continue on the next page

**Below are four questions about screening for breast cancer, so called mammography.**

**11. Are you confident that you know why women aged 40–74 are invited to regular screening for breast cancer?**

[ ] Yes

[ ] No

**12. Have you attended screening for breast cancer, a so called mammogram examination?**

[ ] Yes, no more than 2 years ago

[ ] Yes, more than 2 years ago

[ ] No

[ ] I don’t know

NB! The questions below (13 and 14) are **not** to be completed if you replied “Yes, no more than 2 years ago” to question 12.

**13. For what reasons have you missed out on screening for breast cancer? You can indicate several alternatives.**

[ ] I was afraid to find out that I have cancer

[ ] I don’t believe that cancer happens to me

[ ] I don’t care if I have cancer

[ ] I don’t feel worthy of using healthcare resources

[ ] I don’t have time, due to my addiction

[ ] I prioritized other things

Please specify:______________________________

[ ] I have bad experiences of healthcare services

[ ] I feel persecuted/stigmatized when I visit healthcare services

[ ] I feel that the screening procedure in itself is uncomfortable

[ ] I haven’t received an invitation

[ ] I didn’t comprehend the invitation

[ ] I forgot, or missed the appointment by mistake

[ ] I didn’t have the opportunity to prepare (e.g. maintain hygiene)

[ ] I couldn’t afford transport

[ ] I didn’t want to attend while under the influence

[ ] Other:_______________________­­­­­­­­­­___________________

**14. Is there anything that could make it easier for you to attend cervical cancer screening? You can indicate several alternatives.**

[ ] Social or psychological support

[ ] Practical support, to remember the time and place of appointments

[ ] The opportunity to attend screening at a needle exchange clinic, OST clinic or other addiction care service

[ ] The opportunity to attend screening at a special clinic for women with drug dependency

[ ] Digital invitations, instead of in the mail

[ ] Better invitation design
Please explain how:______________________________

[ ] Other:______________________­­­____________________

The questionnaire is finished. Thank you for participating!
